# Impact of the COVID-19 Pandemic on Gyne-Oncological Treatment—A Retrospective Single-Center Analysis of a German University Hospital with 30,525 Patients

**DOI:** 10.3390/healthcare10122386

**Published:** 2022-11-28

**Authors:** Sebastian Griewing, Matthias Kalder, Michael Lingenfelder, Uwe Wagner, Niklas Gremke

**Affiliations:** 1 Department of Gynecology and Obstetrics, University Hospital Marburg, Philipps-University Marburg, Baldingerstraße, 35043 Marburg, Germany; 2 Institute for Healthcare Management, Chair of General Business Administration, Philipps-University Marburg, Universitätsstraße 24, 35037 Marburg, Germany

**Keywords:** COVID-19, pandemic, gyne-oncology, maximum service supplier, university hospital

## Abstract

The study pursues the objective of drawing a comparison between the data of gyne-oncology, gynecology, and obstetrics patient collectives of a German university hospital regarding the progression of patient number and corresponding treatment data during the five-year period of 2017–2021 to assess the impact of the COVID-19 pandemic on gyne-oncological treatment. Descriptive assessment is based on data extracted from the database of the hospital controlling system QlikView^®^ for patients hospitalized at the Department of Gynecology and Obstetrics of Marburg University Hospital. Gynecology and gyne-oncology experience a maintained decline in patient number (n_Gynecology_: −6% 2019 to 2020, −5% 2019 to 2021; n_Gyne-Oncology_: −6% 2019 to 2020, −2% 2019 to 2021) with varying effects on the specific gyne-oncological main diagnoses. Treatment parameters remain unchanged in relative assessment, but as gyne-oncology constitutes the dominating revenue contributor in gynecology (35.1% of patients, 52.9% of revenue, 2021), the extent of the decrease in total revenue (−18%, 2019 to 2020, −14%, 2019 to 2021) surpasses the decline in patient number. The study displays a negative impact on the gynecology care situation of a German university hospital for the entire pandemic, with an even greater extent on gyne-oncology. This development not only endangers the quality of medical service provision but collaterally pressurizes gynecology service providers.

## 1. Introduction

The COVID-19 pandemic and concomitant measures have not only taken their toll on sociocultural life but exerted pressure of previously unknown dimensions on efficient and effective medical care. Since the pandemic outbreak, the Coronavirus Disease 2019 (COVID-19) has dominated health research to cover and analyze the effects on medical care in every detail. Globally, the pandemic has put health systems to the test of their resilience and uncovered their weak points at a cost in human and monetary terms [1]. Two and a half years of the pandemic have left their mark on German healthcare provision with a total of 28,180,861 cases, 195,698 intensive care unit (ICU) treatments, and 141,105 confirmed deaths [2]. The strained resilience of many health systems has resulted in shortcomings in a variety of care sectors covering the whole spectrum of acute to elective medicine and led to deleterious effects on the treatment of many disorders [3,4,5]. Healthcare systems, including the German one, have navigated through the unknown pandemic dynamics by applying predictive modeling and policymaking that included distinct restrictions on medical care [6,7]. Naturally, this also tested the gynecological and obstetric care infrastructure for its resilience [8].

Taking gynecology into consideration, the swift disease spread in the early beginnings of the pandemic in 2020 led to an almost shock-induced paralysis of medical caretaking, leaving an inexplicable patient number decline in both the out- and in-patient sectors [9,10,11]. On the contrary, the literature suggests that obstetrics and gynecology care can be maintained during a pandemic situation without jeopardizing care quality [12,13]. Gyne-oncology has moved into focus on being particularly endangered of negative impact on its care situation [14,15,16]. As gyne-oncology not only includes breast cancer, the most common type of female malignant neoplasm, but also accounts for 39.2% of overall malignancies and 27.7% of cancer deaths of German women, it is of utmost societal interest to comprehend the development of gyne-oncology care against the backdrop of the COVID-19 pandemic [17]. Nevertheless, most articles only assess the progression of singular or few diseases, limit their study design to short time periods of a few months, or neglect subsequent impact on an economic level [14,15,16]. This insufficient reporting situation called for a more nuanced scientific assessment of the impact of the COVID-19 pandemic on gyne-oncological treatment that takes the whole treatment spectrum and longer time period into consideration and, furthermore, broadens the horizon of observation to health policymaking and economics.

Therefore, this study aims at assessing the treatment data of gynecology, obstetrics, and gyne-oncology patient collectives of a German university hospital to compare the entire pandemic period of 2020–2021 with the previous years of 2017–2019 in order to shed light on the advancement of the care situation. It is the intention to reveal the progression of the chronological development of patient numbers during the first two years of the COVID-19 pandemic and identify related effects on treatment parameters with an outlook on economic efficiency to uncover shortcomings in gynecological care with a specific focus on gyne-oncological disorders.

## 2. Patients and Methods

### 2.1. Data Extraction

Data retrieval is based on the database provided by the hospital controlling system QlikView^®^ technology (Qlik, King of Prussia, PA, USA). Corresponding data were extracted in a retrospective manner. Time horizon of data generation was set for the five-year period from 1 January 2017 to 31 December 2021. Initial extraction was performed for all patients admitted to the Department of Gynecology (patient collective n_Gynecology_) and Obstetrics (patient collective n_Obstetrics_) of Marburg University Hospital during the observation period. Furthermore, based on ICD codification (ICD-10-GM 2022, International Statistical Classification of Diseases and Related Health Problems, 10th Revision, German Modification 2022), corresponding data were compiled for the sub-ordinate patient collective of cases with malignant gyne-oncological main diagnosis (patient collective n_Gyne-Oncology_) and decomposed regarding each entity (C50-58). To comply with national ethics and compliance standards, data were fully anonymized before editing to prohibit the identification of individuals.

### 2.2. Methodology

Primary and secondary diagnoses based on ICD classification as well as the length of stay (LoS), the patient clinical complexity level (PCCL), and case mix index (CMI) are used as parameters for comparison of patient collectives and malignant gyne-oncological main diagnoses. Furthermore, the revenue of the Department of Gynecology in absolute figures was captured for each year of observation. Data assessment is based on descriptive statistics methods using Excel^®^ (Version 16.65, Microsoft Corporation, Redmond, WA, USA). Therefore, relative comparisons (change in %) for consecutive years (i.e., 2018 to 2019) and pandemic years to the pre-pandemic baseline of 2019 (i.e., 2019 to 2020 or 2019 to 2021) were calculated for the parameters. Moreover, graphical visualization of the monthly progression of patient hospitalizations of the gynecology, obstetrics, and gyne-oncology patient collectives as well as C50 malignant neoplasm of the breast main diagnosis in combination with the absolute number of admitted COVID-19 patients at Marburg University Hospital was illustrated using PowerPoint^®^ (Version 16.66.1, Microsoft Corporation, Redmond, WA, USA) and combined with dates of pandemic events and measures taken by the German Government.

### 2.3. Patients

Treatment data of a total n_Total_ = 30,525 patients of the Department of Gynecology and Obstetrics of Marburg University Hospital were extracted from QlikView^®^ for the past five years. The sub-sample of the gyne-oncology patient collective (n_Gyne-Oncology_ = 3359) divides into eight sub-groups (C50–C58). The gyne-oncological main diagnoses of C57 malignant neoplasm of other and unspecified female genital organs and C58 malignant neoplasm of the placenta were excluded from detailed illustration based on the low overall patient number and for C55 malignant neoplasm of the uterus, part unspecified there has not been any registered case within the observation period.

## 3. Results

### 3.1. Overview of the Gynecology, Obstetrics, and Gyne-Oncology Patient Collectives (2017–2021)

The obstetrics patient collective illustrates a yearly relative increase (n_Obstetrics_ = 20,972) in the second year of the pandemic in comparison to the first year and the pre-pandemic baseline of 2019 (n_Obstetrics2021_ = 5000; +20% 2019 to 2021; +10% 2020 to 2021). Accordingly, the total patient number of gynecology represents a relative increase for the second to first pandemic year and a relative decrease in comparison to the pre-pandemic baseline n_Gynecology2021_ = 1994; −5% 2019 to 2021, +1% 2020 to 2021). The sub-sample of patients with malignant gyne-oncological main diagnosis depicts a relative decline for the first and second pandemic year in comparison to the pre-pandemic baseline of 2019 (n_Gyne-Oncology_ = 3359; −6% 2019 to 2020, −2% 2019 to 2020; +3% 2020 to 2021). The corresponding development of the specific main diagnoses C50-C58 is represented in Table 1.

### 3.2. Detailed Results of the Treatment Data of the Gyne-Oncology Patient Collective (2019–2021)

The development of treatment data of the gyne-oncology patient collective (C50–C56) for pre-pandemic (2019) as well as pandemic years (2020–2021) is summarized in Table 2. Furthermore, the equivalent data of gynecology (GYN, n_Gynecology_) and obstetrics (OB, n_Obstetrics_) patients are included for comparability reasons. 

C50 depicts the highest relative share in total gyne-oncological case count by 69% while contributing 49% to the gyne-oncological patient collective’s revenue generation in 2021. Furthermore, the second largest revenue contributor can be seen in C56 by 25.3% while accounting for 13.4% of overall gyne-oncology patients. Longest LoS can be identified for C52 (13.6 days, 2021), followed by C56 (12.9 days, 2021), and, on the other hand, C50 represents the lowest equivalent value (4.7 days, 2021). The gyne-oncological patient collective demonstrates that 75.9% of all patient stays are completed within the normal LoS limits according to the current German DRG (German Diagnosis Related Groups, Version 2022) catalog. In gyne-oncology, C50 and C51 depict the longest and shortest (90% and 73%, 2021) equivalent values. Regarding standardized health economic parameters of CMI and PCCL, C56 and C50 depict the highest and lowest averages for gyne-oncology while the comparable gynecology and obstetrics patient collective represents even lower values (C50_2021_: CMI = 1.2, PCCL = 0.4; C56_2021_: CMI = 3.2, PCCL = 2.5; GYN_2021_: CMI= 1.1, PCCL 0.4; OB_2021_= CMI = 0.6, PCCL = 0.4). Overall, gyne-oncology amounts to 35.1% (GYN*^1^) of total gynecology patients and contributes 52.9% of total revenue generation in the respective department in 2021 (GYN*^2^). Furthermore, the total revenue of the Department of Gynecology declines during the pandemic (−18%, 2019 to 20 and −14%, 2019 to 21) after preceding years of growth (+7% 2017 to 18, +9% 2018 to 19).

### 3.3. Results of the Chronological Visualization of the Patient Collectives (2019–2021)

**Figure 1:**
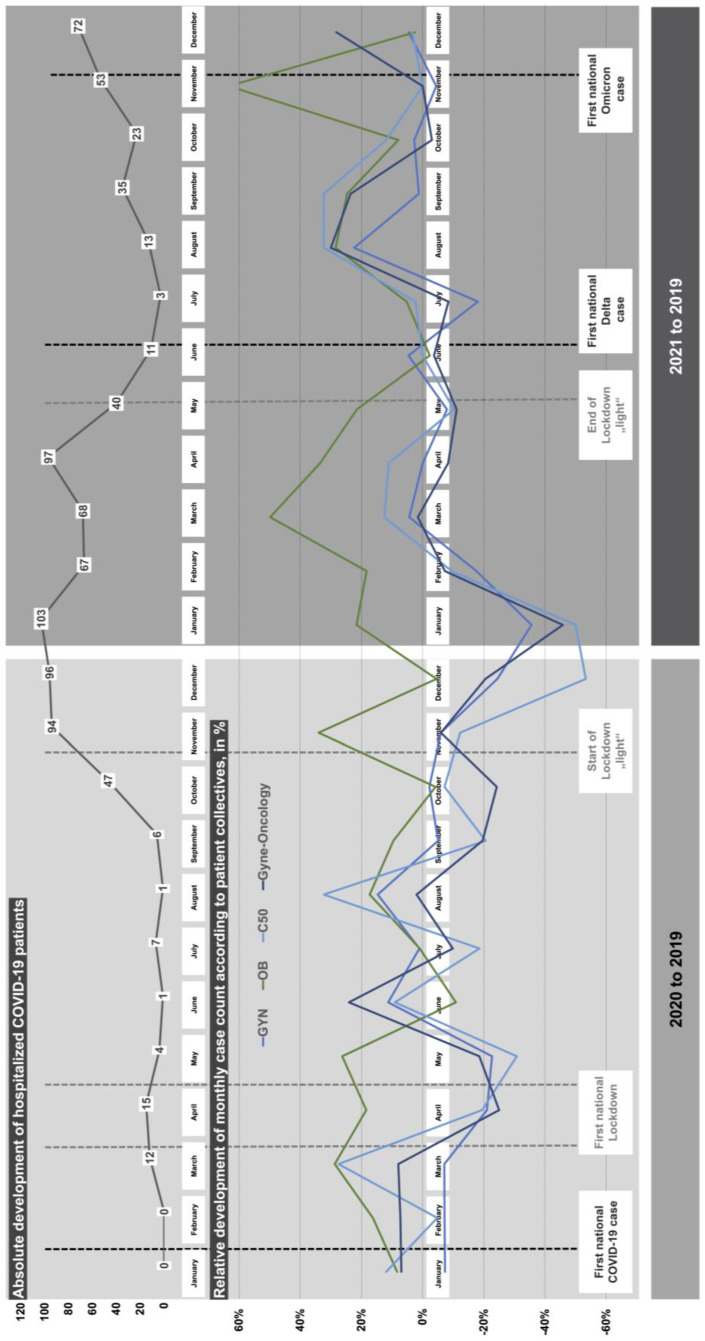
Monthly relative case development of the gynecology, obstetrics, and gyne-oncology patient collectives (2019–2021) and absolute number of hospitalized COVID-19 patients at Marburg University Hospital.

The monthly caseload in 2019 of the gynecology, obstetrics, gynecology-oncology patient collectives, as well as the sub-group of C50 main diagnosis, was used for the visualization of the relative change in comparison to the pandemic years of 2020 and 2021 (see Figure 1). Table 3 illustrates the according monthly change in percent. Pandemic measures enforced by the German government and national lockdown time periods are included in Figure 1 and Table 3.

## 4. Discussion

Measures enforced by health policymaking, i.e., restrictions on operating room capacities or clearance of hospital beds to free up capacities for expected hospitalizations of COVID-19 patients, have shaken well-established structures and processes of caretaking in hospitals and rendered timely and effective care impossible at times [3,18,19]. The cardiovascular burden of the pandemic, for example, was extensively discussed in the latest literature [20,21]. Short-term treatment, post-vaccination, and long-term complications of SARS-CoV-2, i.e., in the form of deep vein thrombosis, pulmonary embolism, myocarditis, or even sudden cardiac death, have been identified to contribute to an increased cardiovascular threat to the population [22,23,24]. On the other side, an adverse development of cardiac emergency care has been reported with inexplicable low numbers, i.e., of myocardial infarction, during phases of high COVID-19 incidence [25]. Further articles show that this development does not only apply to medicine but also to emergency and elective surgery of all kinds [4,5,26,27]. Di Martino et al. provide an interesting study that connects the fields of oncology with surgery by assessing the variations in elective oncological surgery on colorectal and breast cancer during the first pandemic year of 2020 in comparison to the previous years of 2018 and 2019. Therefore, they have evidenced a significant decrease in colorectal and breast cancer interventions by 35.71% and 10.36% in the Abruzzo region in Italy [28]. Our study expands on these previous findings by diving deeper into the gynecology and gyne-oncology disease spectrum as current literature limits assessment to singular or few diseases, shorter time periods and, furthermore, it neglects the influence on an economic level, a gap this article aims to fill in [14,15,16].

### 4.1. Comparison of the Patient Collectives

The data confirm that obstetric care remains largely unaffected by the effects of COVID-19 in terms of patient number progression and extends this finding to 2021, the second year of the pandemic [11,29]. The pre-pandemically prevailing development of increasing patient numbers (+12%; 2018 to 2019) continues for the years 2020 (+10%, 2019 to 2020) and 2021 (+10%, 2020 to 2021). This progression is supposed to trace back to the upholding trend of the continuous centralization of the care infrastructure due to the closure of peripheral service providers in Germany that takes root in critical economic efficiency and challenging insurance burden in obstetric care.

On the other hand, the gynecology patient collective highlights a worrisome progression of in-patient cases for the catchment area of Marburg University Hospital that expands the previously known observations of the first pandemic year [9,10]. While current literature predominantly identified a decrease in gynecology admissions during the first phase of high national COVID-19 incidence, this study extends this observation to two years of the pandemic follow-up assessment [13]. After two years of consecutive growth (+6%, 2017 to 2018; +9%, 2018 to 2019), the gynecological medical service provision was shaken by the COVID-19 pandemic in 2020 (−6%, 2019 to 2020) and, unfortunately, this development holds on for the second pandemic year of 2021 (−5%, 2019 to 2021).

Even worse, this harmful observation is mirrored in overall malignant gyne-oncology patient number (−6%, 2019 to 2020; −2%, 2019 to 2021), contradicting a preceding pre-pandemic phase of growth (+1%, 2017 to 2018; +7% 2018 to 2019) that was predominantly based on a continuous expansion of the catchment area of the Department of Gynecology of Marburg University Hospital and the closure of peripheral gynecological service providers in the past years. This expands the observations of Di Martino et al. of a decrease in admissions for elective breast cancer surgery to the entire gyne-oncological range of treatment and the second year of the COVID-19 pandemic [28]. Keeping in mind that gyne-oncology accounts not only for 35.1% of overall gynecology patients (GYN*^1^) but also for 52.9% of the department’s revenue generation (GYN*^2^), the feared medical damage relating to diagnostics and treatment delays, failing screening procedures, and related worsened treatment outcome is accompanied with a possibly fierce recess in the economic performance. While the data expand previous observations by confirming the total patient number of gyne-oncology to decrease for the whole COVID-19 pandemic, it depicts a varying effect on the specific malignant main diagnoses [9,10,14,30].

### 4.2. Discussion of the Chronological Visualization of the Patient Collectives

The visualization of the relative monthly development of patient numbers leads to the following observations that expand the previous scientific status quo [9,10,12,14,16,30]. Obstetric care appears to not have been affected to a large extent by the effects of the COVID-19 pandemic in terms of admitted cases at Marburg University Hospital, this expands the finding to the second year of the pandemic [12]. Vice versa, gynecology patient number reacts to rising regional COVID-19 case numbers during the first and second lockdowns with an approximate latency of a month. The amount of hospitalized COVID-19 patients and the intensity of decline in patient numbers for gynecology and gyne-oncology patient collectives turned out to be higher during the second lockdown “light”, lasting for a considerably longer time [9,10]. The lockdown “light” was initiated by the German government as a reaction to the once again accelerated increase in national and regional COVID-19 case numbers in September 2020. This observation extends previous findings of Jacob et al. for gynecological cancer to an in-patient environment and the time horizon of the entire first two years of the pandemic in Germany [9,10]. Furthermore, the relative negative impact on patient number for gyne-oncology surpasses the corresponding intensity for gynecology, with malignant neoplasm of the breast experiencing an even greater decline. Despite sustained high levels of hospitalized COVID-19 patients at Marburg University Hospital from February to May 2021, the patient collectives return and surpass pre-pandemic care levels from March 2021 onwards, followed by a phase of recuperation in August and September 2021. Lastly, the confirmation of the first national Delta variant in June 2021, that is followed by another swift increase in hospitalized COVID-19 patients, as well as the first official Omicron variant registration in November 2021, appear to have no measurable effect on the care situation for gynecology and gyne-oncology, while higher morbidity and mortality for the Delta variant [31,32,33,34] as well as faster spread due to higher contagiousness for the Omicron variant [35,36] have widely been discussed. To our knowledge, there has not been any literature so far that monitors the possible impact of new virus variants on gynecology or gyne-oncology care.

### 4.3. Discussion of the Treatment Data of the Gyne-Oncology Patient Collective

In general, published data on gynecologic treatment with an outlook on economic efficiency present to be very superficial and scarce, and therefore, these results expand the knowledge on the challenging economic state of gynecology and obstetrics care [37,38]. Only contributing about a third (GYN*^1^ = 35.1%) to the department’s patient number but providing 52.9% of overall revenue generation (GYN*^2^) sets apart the importance of gyne-oncology for the economically sound management control and realizing a possible break-even as a gynecologic maximum service provider. Furthermore, the illustrated averages of treatment parameters for 2021 of the Department of Gynecology and Obstetrics underline how critical economic efficiency or even cost coverage can be with comparably low average CMI and PCCL (GYN: CMI = 1.1, PCCL = 0.4); OB: CMI = 0.6, PCCL: 0.4). This finding is accompanied with a low average length of stay (GYN: 4.6 days; OB: 3.9 days) leading to a high daily case turnover. In the case of Marburg University Hospital, the majority of patient stays were concluded below the maximum DRG-based upper LoS limits (GYN: 96.3%; OB: 97%) nonetheless. Consequently, the extent of the decrease in total revenue of the Department of Gynecology during the pandemic years (−18%, 2019 to 2020, −14%, 2019 to 2021) turns out to be even higher and clearly surpasses the intensity of decline in number of patients (GYN: −6%, 2019 to 2020; −5% 2019 to 2021; Gyne-Oncology: −6%, 2019 to 2020; −2% 2019 to 2021). Although combining the assessment of the chronological advancement of gyne-oncological patient numbers appears to be unusual at first glance, we identify it to be very useful for understanding underlying external factors for case development. The literature presents to be very scarce on these issues, and therefore, the data analysis sheds light on the vicious cycle of centralization and increasing case turnover in combination with decreasing economic efficiency [37,38]. In their review article, Haldane et al. illustrate how the COVID-19 pandemic has tested health systems’ resilience on a global scale and how the shock manifested each system’s weak points. Furthermore, the authors point out that the uncovering of a system’s failure also provides the base to build up its resilience [1]. The lessons from COVID-19 must be learned not only for entire health systems but also for regional care networks or individual medical fields [6]. The presented article provides an outlook on the critical economic efficiency of gynecological care, which is based on low and gradually decreasing CMI and PCCL to a great extent. It points toward the high dependence on gyne-oncology treatment as the main revenue contributor using the example of Marburg University Hospital and illustrates how the decline in admissions during COVID-19 not only endangered the quality of medical service provision in the regional network but could also have collaterally pressurized the gynecology service provider on an economic level. The ongoing vicious cycle consisting of rising patient numbers on the one side and decreasing case-related economic efficiency on the other side must move into the focus of health policymaking to build up the resilience of gynecology care as an individual medical field, also keeping in mind its important role for the entire health system’s resilience.

### 4.4. Limitations and Future Research

The study is built upon the analysis of a single center data evaluation of Marburg University Hospital, a maximum care provider in Germany. This limits the transfer and generalizability of the results to other settings and health systems. Renumeration for in-patient treatment in the German healthcare system is based on Diagnosis Related Groups (German DRG), and therefore, data comparability may be best for other DRG-based health systems. Furthermore, the German system includes comprehensive health coverage with compulsory public health insurance for each resident and an opt-out option for private health insurance for high-income individuals. Interpretation of the manuscript’s results is limited to this particular health system context.

Although due to its regional infrastructure, the catchment presents to be rather rural, Marburg-Biedenkopf county and its surroundings offer a well-balanced socio-economic environment that compares to German standards. The high degree of medical care centralization at Marburg University Hospital due to missing peripheral service providers of considerable size provides a sound base to conduct regional health research assessments. Its retrospective design restricts the analysis to the application of descriptive statistical assessment of already finished patient stays coded in the QlikView^®^ database. Therefore, the study only includes in-patient care. External effects of fraudulent coding, misdiagnosis, or changes in ICD classification remain possible. It is important to note that the article is built upon an iterative and explorative top-down approach to decompose the gynecological, obstetrics, and gyne-oncological disease spectrum of Marburg University Hospital in order to identify the related impact on the care situation during the COVID-19 pandemic. Due to the explorative nature of data evaluation, the findings are restricted to descriptive assessment so far. Future research efforts must expand on the findings of this article by adding further variables into a study design (i.e., mortality, relapse rate, vaccination status, treatment complications, etc.) to allow for the evaluation of the statistical significance of the described developments. Corresponding tests in the form of time and trend or comparative analysis would also bear the potential to investigate the causality behind the identified variations. Moreover, it would be beneficial to extend future studies to multicenter comparison of data to confirm the findings in urban areas and an outpatient environment. Regarding the representation of treatment data, the study provides an outlook on economic efficiency. This assessment is limited to standardized health economic parameters and revenue-based valuation, leaving out an evaluation of cost and profit performance. Further analysis should combine the data from cost- and profit-oriented perspectives to evaluate the economic efficiency of discussed diseases in a multi-faceted manner. Therefore, the design provides a considerable number of limitations.

## 5. Conclusions

The study draws a comparison between the gynecology, obstetrics, and gyne-oncology patient collectives of Marburg University Hospital and related treatment data to identify the impact of the COVID-19 pandemic on gyne-oncological treatment and to provide an outlook on the economic efficiency of care. It contributes to previous scientific findings by expanding the time horizon of observation, covering the whole gyne-oncological disease spectrum, providing an illustration of the chronological development of patient numbers to the second year of COVID-19, and including an economic perspective. Up to now, studies have limited their analyses to shorter time periods of the pandemic and single disorders or disease groups within gynecology and obstetrics. Complementing international studies, the caseload of the gynecology patient collective shows a decrease in the observation period, while obstetric care remains largely unaffected in terms of patient number. The findings suggest that this harmful observation turns out to be even greater in the gyne-oncological care situation as gyne-oncology constitutes the dominating revenue contributor in gynecology (35.1% of patients, 52.9% of revenue, 2021), and therefore, the extent of decrease in total revenue (−18%, 2019 to 2020; −14%, 2019 to 2021) surpasses the decline in patient number. Therefore, the study expands the illustration of patient number development with an outlook on economic efficiency that proves to be important for data interpretation. Imposed pandemic measures had a visibly negative impact on in-patient treated gynecology patients that presents to be even worse for gyne-oncology. In past years, the combination of rising case turnover and decreasing case-related economic efficiency built up vulnerability on medical and economic levels for gynecological care in the case of Marburg University Hospital, a crucial weak point that was uncovered by the pandemic. Overall, this may have led to a pressurized care situation for gynecology and obstetrics care providers during the COVID-19 pandemic, not only on a medical but also on an economic level. Health policymaking needs to utilize the lessons learned from the system’s failure and use them as a base to build up resilience, not only for gynecology as a singular medical field but also for the entire healthcare system.

## Figures and Tables

**Table 1:** Absolute and relative development of patient number of gynecology, obstetrics, and gyne-oncology patient collectives.

Patient Number, 2017–2021	Absolute Number					Relative Change in %			Share of Overall Gyne-Oncology Cases, in %
	2017	2018	2019	2020	2021	18 to 19	19 to 20	19 to 21	
Main Patient Collectives									
n_Gynecology_	1767	1879	2045	1918	1944	9%	−6%	−5%	n.a.
n_Obstetrics_	3565	3699	4150	4558	5000	12%	10%	20%	n.a.
n_Gyne-Oncology_	652	658	702	662	685	7%	−6%	−2%	n.a.
Gyne-Oncology Patient Collective									
C50 Malignant neoplasm of the breast	407	424	461	429	469	9%	−7%	2%	68.5%
C51 Malignant neoplasm of the vulva	36	33	40	47	26	21%	18%	−35%	3.8%
C52 Malignant neoplasm of the vagina	5	3	5	7	7	67%	40%	40%	1.0%
C53 Malignant neoplasm of the cervix	45	46	39	32	30	−15%	−18%	−23%	4.4%
C54 Malignant neoplasm of the corpus uteri	80	70	67	61	57	−4%	−9%	−15%	8.3%
C56 Malignant neoplasm of the ovary	72	81	85	73	91	5%	−14%	7%	13.3%
C57 Malignant neoplasm of other and unspecified female genital organs	3	1	5	6	2	400%	20%	−60%	0.3%
C58 Malignant neoplasm of the placenta	4	0	0	7	3	n.a.	n.a.	n.a.	0.4%

n.a. = not applicable, red color indicating a decrease, green color indicating an increase.

**Table 2:** Treatment data of the patient collectives and gyne-oncological sub-groups.

Treatment Parameters		C50			C51			C52			C53			C54			C56			GYN	OB
2019–2021		2019	2020	2021	2019	2020	2021	2019	2020	2021	2019	2020	2021	2019	2020	2021	2019	2020	2021	2021	2021
Share of overall oncological patient number, in %		66.1%	66.1%	69.0%	5.7%	7.2%	3.8%	0.7%	1.1%	1.0%	5.6%	4.9%	4.4%	9.6%	9.4%	8.4%	12.2%	11.2%	13.4%	35.1% (GYN*^1^)	
Share of overall revenue in oncology, in %		48.9%	49.4%	49.1%	5.2%	8.8%	6.7%	1.4%	2.2%	1.4%	7.9%	6.6%	5.7%	13.4%	11.9%	11.8%	23.2%	21.3%	25.3%	52.9% (GYN*^2^)	
Average LoS, in days		4.8	5.1	4.7	9.1	10.8	15.7	12.6	12.1	13.6	9.5	9.5	9.5	10.3	9,6	10.5	13.6	13.4	12.9	4.6	3.9
LoS-D, in %	Normal LoS	90%	91%	90%	79%	80%	73%	80%	86%	57%	85%	84%	80%	83%	92%	82%	87%	85%	89%	75.9%	74.5%
	Below limit LoS	6%	6%	7%	18%	13%	12%	20%	14%	14%	13%	13%	17%	9%	5%	13%	6%	7%	4%	20.3%	22.5%
	Above limit LoS	4%	3%	3%	3%	7%	15%	0%	0%	29%	3%	3%	3%	8%	3%	5%	7%	8%	7%	3.7%	3.0%
CMI		1.5	1.3	1.2	1.8	2.0	2.9	2.8	2.3	2.2	2.8	2.3	2.2	2.8	2.1	2.4	3.8	3.2	3.2	1.1	0.6
PCCL		0.5	0.5	0.4	0.8	1.2	1.5	1.1	1.2	1.1	1.1	1.2	1.1	1.6	1.1	1.5	2.5	2.1	2.5	0.4	0.4
Average number of SD		2.0	2.8	4.7	3.6	7.8	11.3	3.9	6.3	8.5	3.9	6.3	8.5	6.2	6.3	11.1	8.0	9.4	14.9	5.3	5.5

LoS = length of stay, LoS-D = length of stay distribution of all patients, SD = secondary diagnoses, CMI = case mix index, PCCL = patient clinical complexity level, GYN*^1^ = gyne-oncology share of total gynecology patients in %, GYN*^2^ = gyne-oncology share of total revenue in %.

**Table 3:** Overview of the monthly relative case development of the gynecology, obstetrics, and gyne-oncology patient collectives (2019–2021) at Marburg University Hospital.

Compared Time Periods		Months	GYN	OB	C50	Gyne-Oncology
			Relative change, in %			
2019 to 2020	27 January 2020 First national COVID-19 case	January	−7%	8%	12%	7%
		Feburary	−7%	16%	−5%	7%
	22 March 2020 Start of “hard” Lockdown	March	−7%	29%	28%	8%
		April	−21%	18%	−19%	−25%
	03 May 2020 First relaxations of contact measures	May	−23%	26%	−31%	−19%
		June	11%	−11%	9%	24%
		July	1%	1%	−19%	−10%
		August	15%	17%	32%	2%
		September	−5%	10%	−21%	−20%
		October	−2%	−4%	−7%	−24%
	Start of lockdown “light”	November	−6%	34%	−12%	−6%
		December	−24%	−5%	−53%	−21%
2019 to 2021	Initiation of national vaccination program	January	−36%	22%	−50%	−46%
		February	−18%	18%	−10%	−7%
		March	4%	50%	13%	2%
		April	0%	34%	11%	−8%
	End-of-contact measures	May	−8%	21%	−10%	−11%
	First official delta variant case	June	5%	−2%	0%	−4%
		July	−18%	5%	2%	−8%
		August	22%	28%	32%	30%
		September	1%	25%	32%	24%
		October	3%	8%	12%	−3%
	First official omicron variant case	November	−4%	63%	0%	0%
		December	5%	2%	3%	28%

Red color indicates a decrease, green color indicates an increase, light grey shadowing for the 2019 to 2020 comparison, dark grey shadowing for the 2019 to 2021 comparison, blue shadowing indicates the lockdown periods.

## Data Availability

The datasets generated during and analyzed during the current study are available from the corresponding author upon reasonable request.
